# Application of metabolomics and network analysis to reveal the ameliorating effect of four typical “hot” property herbs on hypothyroidism rats

**DOI:** 10.3389/fphar.2022.955905

**Published:** 2022-08-25

**Authors:** Yang-Yang Wang, Yan-Ping Sun, Bing-You Yang, Qiu-Hong Wang, Hai-Xue Kuang

**Affiliations:** ^1^ Key Laboratory of Basic and Application Research of Beiyao (Heilongjiang University of Chinese Medicine), Ministry of Education, Harbin, Heilongjiang, China; ^2^ School of Traditional Chinese Medicine, Guangdong Pharmaceutical University, Guangzhou, Guangdong, China

**Keywords:** Typical “hot” property herbs, metabolomics, hypothyroidism, network analysis, metabolic mechanism, cold-hot medicine properties

## Abstract

Herbs with a “hot” properties are frequently used to treat cold symptoms in TCM. However, the underlying mechanisms of the herbs with “hot” properties on hypothyroidism have not been investigated. This study aimed to explore four typical “hot” and “cold” property herb on hypothyroidism. Firstly, the difference efficacy between the four typical “hot” property herbs and the four typical “cold” property herbs was assessed by physical signs, thyroid function, and the metabolic profile using multivariate statistical analysis. The influence of the four typical “hot” property herbs on hypothyroidism was validated pathologically. The impact mechanism of the four typical “hot” property herbs on hypothyroidism was investigated through a metabolomics method combined with network analysis. Na^+^/K^+^-ATP, ACC1 enzyme, UCP-1, and the PI3K-Akt pathway were used to confirm the metabolite pathways and target-associated metabolites. The results showed that the four typical “hot” property herbs could significantly improve physical signs, thyroid function, and the metabolic profile in hypothyroidism rats, the four typical “cold” property herbs did not show any benefit. Moreover, the four typical “hot” property herbs could improve lipid metabolism, energy metabolism, and thyroid hormone levels by the PI3K-Akt signaling pathway, Ca^2+^- AMPK signaling pathways, purine metabolism, and tryptophan metabolism. Additionally, the levels of UCP-1, Na+/K + -ATP enzyme, and ACC1 were ameliorated by the four typical “hot” property herbs in hypothyroidism rats. Therefore, a metabolomics strategy combined with network analysis was successfully performed and interpreted the mechanism of the four typical “hot” property herbs on hypothyroidism based on the theory of “cold and hot” properties of TCM well.

## Introduction

Hypothyroidism is on of the most common thyroid diseases affecting people worldwide, particularly during pregnancy and childhood (Taylor et al., 2018). Obesity, cold limbs, depression, hyperlipidemia, negative emotions, reduced lipolysis, and gluconeogenesis are the most essential clinical manifestations (Meier and Kaplan, 2002). Currently, thyroid hormone replacement therapy is the main treatment for hypothyroidism (Clarke and Kabadi, 2004). However, during hypothyroidism therapy, the level of thyroid hormones is difficult to regulate, and over-medication was prevalent, increasing the risk of cardiovascular disease (Flynn et al., 2010), osteoporosis (La Vignera et al., 2008), and subclinical liver damage (Beckett et al., 1985). Therefore, there is an urgent need to seek new strategies for the management of hypothyroidism. According to the traditional Chinese medicine (TCM) theory, hypothyroidism is classified as a “cold” syndrome, and “treating cold syndrome with hot herbs and treating heat syndrome with cold herbs” is a fundamental medication principle of Chinese medicine (Xiao et al., 2017). The “cold and hot” properties of TCM refer to the properties that can cause a certain type of reaction in the body, and are used to treat diseases. However, pharmacology is modern science that studies the regularity of interaction between drugs and the body and the mechanism of the drug effect. Although we have reports suggesting that the “hot” drugs have a significant improvement influencing hypothyroidism (Cheng et al., 2016), there are no reports concerning the typical “hot and cold” property herb influencing hypothyroidism. Therefore, it is necessary to further study the effects of the four typical “hot” property herbs and four typical “cold” property herbs on hypothyroidism to provide a basis for treating hypothyroidism, as well as reveal the scientific essence for the medication principle.

TCM has been applied in preventing and treating the diseased for thousands of years (Feng et al., 2006). Complex-component, multi-target, and multi-path are the typical characteristics of Chinese herbs, which can be used to holistically treat diseases (Pan et al., 2020). One of the major challenges facing TCM is explaining the mechanisms underlying the efficacy of medicines used in TCM (Peng et al., 2018). Therefore, a novel research method consistent with TCM characteristics is crucial. Metabolomics is a high-throughput qualitative and quantitative analysis approach focusing on all small endogenous molecule metabolites in the body to reveal the pathological and physiological status at the overall level (Carneiro et al., 2019). Metabolomics with the systematic biological characteristics is consistent with the theory of TCM and provides a powerful tool for the research on TCM (Cheng et al., 2018). Recently, the network pharmacology approach offered a new understanding of Chinese medicine research from a system perspective and at the molecular level by predicting the interactions between multiple targets of compounds in herbs and/or multiple genes related to diseases (Fang et al., 2017). The metabolic process of the body is mainly catalyzed by enzymes, which are inseparable (metabolites are regulated by proteins, and the activity of proteins is also changed by metabolites, such as described in KEGG). Metabolomics can be used to identify specific molecular markers in certain physiological and pathological conditions (Barnes et al., 2016). Network pharmacology emphasizes the regulation of multiple targets by signal pathways to improve the therapeutic effect of drugs and reduce the toxicity and side effects (Yuan et al., 2017), both of them provide insights into the disease at the molecular and systemic levels. These approaches have been widely applied in the assessment of the therapeutic effects and mechanisms of TCM in recent years (Buriani et al., 2012).

Therefore, an integrated metabolomic strategy combined with network analysis methods was applied to reveal the effects of four typical “hot” property herbs and four typical “cold” property herbs on hypothyroidism. According to the records in ancient books of past dynasties, pharmacopoeia of the People’s Republic of China and modern research in the “cold/hot” property, the four typical “hot” property herbs (Jia et al., 2017; Committee, 2020), including *Aconitum carmichaeli* Debeaux (FZ), *Zingiber officinale* Roscoe (GJ), *Cinnamomum cassia* (L.) J. Presl (RG), and *Euodia ruticarpa* (A. Juss.) Benth. (WZY), were selected. Meanwhile, the four typical “cold” property herbs (Wang et al., 2016; Committee, 2020), including *Scutellaria baicalensis* Georgi (HQ), *Coptis chinensis* Franch. (HL), *Gardenia jasminoides* J. Ellis (ZZ), and *Rheum palmatum* L. (DH) were also selected as a contrasting experiment to explore the medication principles of the “cold/hot” property. Finally, the results of biochemistry, metabolomics, and the network analysis were integrated to clarify the effect of the four typical “hot” property herbs on hypothyroidism.

## Materials and methods

### Chemicals and reagents

Acetonitrile (HPLC grade, United States) and formic acid (LC-MS grade, United States) were purchased from Fisher Chemical. The four typical “cold” property herbs: *Scutellaria baicalensis* Georgi (HQ), *Coptis chinensis* Franch. (HL), *Gardenia jasminoides* J. Ellis (ZZ), and *Rheum palmatum* L. (DH), and the four typical “hot” property herbs: *Aconitum carmichaeli* Debeaux (FZ), *Zingiber officinale* Roscoe (GJ), *Cinnamomum cassia* (L.) J. Presl (RG), and *Euodia ruticarpa* (A. Juss.) Benth. (WZY), which were purchased from Beijing Tongrentang Co., Ltd (Beijing, China). The herbs were authenticated by Professor Zhenyue Wang (Heilongjiang University of Chinese Medicine, Harbin, China) and all voucher specimens were preserved at Heilongjiang University of Chinese Medicine.

6-propyl-2-thiouracil (PTU) was purchased from Sigma-Aldrich (Merck KGaA, Darmstadt, Germany, batch Number: BCBR87087), and L-thyroxine (L-T4) was purchased from Aladdin Biochemical Technology Co., Ltd., (Shanghai, China, batch Number: H2014187).

The ELISA kits for rat triiodothyronine (T3, batch Number: C0384010396), thyroxine (T4, batch umber: C0346030349), and thyroid-stimulating hormone (TSH, batch Number: C0373050316) were purchased from Wuhan Huamei Biotechnology Co., Ltd., (Wuhan, China). The ELISA kits for the Na+/K + -ATP enzyme (batch Number: 202109) and Acetyl CoA carboxylase 1 (ACC1, batch Number: 202109) were purchased from Jiangsu Meimian Industry Co., Ltd. (Jiangsu, China).

### Herb extraction

The four typical “hot” property herbs (FZ, GJ, RG, and WZY) and the four typical “cold” property herbs (HQ, HL, ZZ, and DH) were extracted by decocting with water three times [mass (g): volume (v) = 10:1, 8:1, and 8:1; 0.5 h each]. Then, the combined decoctions were concentrated and dried in a vacuum to obtain a FZ/GJ/RG/WZY/HQ/HL/ZZ/DH crude extract.

### Animals

Sprague–Dawley rats (200 ± 10 g, male) were obtained from Liaoning Changsheng Biotechnology Co., Ltd., [Liaoning, China; Certificate No. SCXK (Liao) 2020–0001]. All the rats were housed under a 12 h/12 h light/dark cycle, at a temperature of 20 ± 3°C, with 60% ± 10% relative humidity. They had free access to standard food and sterile-filtered water. All the animal experiments were approved by the Experimental Animal Ethics Committee of the Heilongjiang University of Chinese Medicine and performed in accordance with relevant guidelines.

After adaptive breeding for 1 week, all rats were randomly divided into 11 groups: control group (control, *n* = 7), hypothyroidism model group (Hypo, *n* = 8), positive drug group (Hypo + T4, *n* = 8), and treatment group by the four typical “hot” property herbs, including Hypo + FZ (*n* = 8), Hypo + GJ (*n* = 8), Hypo + RG (*n* = 8), and Hypo + WZY (*n* = 8), as well as the contrasting experimental group by the four typical “cold” property herbs, including Hypo + HQ (*n* = 8), Hypo + HL (*n* = 8), Hypo + ZZ (*n* = 8), and Hypo + DH (*n* = 8). With the exception of the rats in the control group, the hypothyroidism model was established in all rats by intraperitoneal injection PTU (10 mg/kg/d) for 28 days consecutively (Baltaci and Mogulkoc, 2018). The positive drug group was intragastrically administered T4 (0.3 mg/kg/d) from day 14 to the end. The treatment group and contrasting experimental group FZ (7 g/kg/d), GJ (4.7 g/kg/d), RG (2.4 g/kg/d), WZY (2.4 g/kg/d), HQ (4.7 g/kg/d), HL (2.4 g/kg/d), ZZ (4.7 g/kg/d), DH (7 g/kg/d) were respectively continuous intra-gastrically administered for 28 days, respectively (The dose was calculated according to the Chinese Pharmacopoeia).

### Sample collection and preparation

After the last administration, the rats were placed alone in metabolic cages and the collection tube in a box with ice was kept to reduce bacterial contamination, for 12 h urine collection, which was centrifuged for 10 min, at 12,000 rpm. The supernatant was stored at −80°C until analysis. After euthanasia with pentobarbital sodium by intraperitoneal injection, the serum was collected by centrifugation at 3,000 rpm for 10 min and stored at −80°C until further analyses. The live, brown adipose tissue (BAT), the thyroid gland were obtained and fixed in 4% paraformaldehyde for histological examinations. Moreover, approximately 0.1 g of the liver tissue was homogenized at a 0.9% saline solution, which was used to detect the Na^+^/K^+^-ATPase and ACC l activities and frozen at −80°C before use.

The urine sample was mixed with four-fold cold acetonitrile, vortexed for 2 min, and centrifugation at 4°C at 13,000 rpm for 10 min. After high-speed centrifugation, the prepared supernatant was transferred into a fresh 2 ml LC-MS glass vial. In addition, the quality control (QC) sample was prepared by mixing an equal amount of every sample from an identical experiment group. Finally, 2 μL of the supernatant for each sample was injected into the UPLC-Q-TOF/MS system for metabolomic analysis.

### UPLC-Q-TOF/MS analysis

An ACQUITY UPLC system (Waters, Milford, United States) in tandem with a Q-TOF synapt G2-SI mass spectrometer (Waters, Milford, United States) was acquired for LC-MS/MS analyses with an ACQUITY UPLC HSS T3 column (1.8 μm, 2.1 mm × 100 mm, Waters, Milford, United States). The chromatography separation was performed at an ambient temperature of 40°C. The mobile phase included acetonitrile with 0.1 % formic acid (A), and deionized water with 0.1% formic acid (B), and the gradient of eluent was used: 0–15 min, 5%–68 % (A); 15–18 min, 68 %–95 % (A); 18–19 min, 95 %–5 % (A); and 19–20 min, 5 % (A). The flow rate was set at 0.3 ml/min, and the injection volume was 2 µL.

The key parameters of Q-TOF/MS were optimized as follows: scan type: positive and negative, acquire mass over the range 100–1300 Da, scan time: 0.25s, collision energy: 25–35 V, cone voltage: 40 V. the electrospray capillary voltage was 3.2 kV in the positive and negative ionization modes, the ion source temperature was 320°C, and the auxiliary heater temperature was 350°C.

### Data processing and metabolism profile analysis

After the UPLC-Q-TOF/MS analysis, the raw data were imported into the Progenesis QI software (version 2.0, Nonlinear Dynamics, Waters, United States) for peak detection, alignment, deconvolution, and normalization. The resultant data matrices were linked to EZ info (version 2.0, Waters Corporation) for the principal component analysis (PCA) and orthogonal partial least squares discriminant analysis (OPLS-DA). The list of ions that contributed to the grouping was obtained from loading the S-plot and variable importance plot (VIP). The biomarkers were identified by MS/MS fragment ion and accurate mass by searching reliable online biochemical databases such as the Human metabolome database (HMDB) (http://www.hmdb.ca/) and Kyoto Encyclopedia of Genes and Genomes (KEGG) (https://www.kegg.jp/kegg/) in the Progenesis QI software (Ren et al., 2016).

### Hematological analyses, biochemical parameters, and real-time -PCR assay

On day 28, the thyroid tissue was soaked in 4% paraformaldehyde, embedded in paraffin, cut into 5-mm sections, and stained with Hematoxylin and Eosin (H&E). The sections were visualized using light microscopy (Nikon, Tokyo, Japan), and digital images were captured and analyzed.

A commercial ELISA kit (Wuhan Huamei Biotechnology Co., Ltd., Wuhan, China) was used to analyze the levels of T3, T4, and TSH in the serum in accordance with the manufacturer’s protocol. ACC1 and Na^+^/K^+^-ATP enzymes in the liver were detected by a commercial ELISA kit (Meimian, Jiangsu, China) in accordance with the manufacturer’s protocols. The rectal temperature of the rats was measured by an electronic standard rectal thermometer and the bodyweight of the rats was weighed by electronic scales at days 1, 7, 14, and 28.

The extracted total RNA from BAT, the primers designed, and the cDNA synthesis were references reported in the literature, which was performed by Wuhan Servicebio Biotechnology Co., Ltd. (Wuhan, China) (Tian et al., 2021). Primers were specific to the target genes, namely: UCP1: 5′- CGG​GCT​TAA​AGA​GCG​AGA​GG- 3’ (forward, accession no. NM_012682.2); GAPHD: 5′- CTG​GAG​AAA​CCT​GCC​AAG​TAT​G- 3’ (forward, accession no. NM_017008.4). Gene expression was evaluated by the DDCT method with GAPDH as a housekeeping gene.

Assay of the Western blot, as previously reported (Zhang et al., 2021), the levels of PI3K (A4992, Abcam, MA, United States), Akt (Ap1208, Abcam, MA, United States), ULK (A8529, Abcam, MA, United States), IL17 (Ao688, Abcam, MA, United States), P-p38 MAPK (AP0526, Abcam, MA, United States), PPAR-γ (A19676, Abcam, MA, United States), UCP-1 (A5857, Abcam, MA, United States), and β-actin (A8227, Abcam, MA, United States) in the liver were examined by using Western blot.

### Data collection of network analysis

Firstly, the chemical compositions of the four typical “hot” property herbs from the literature (searched from CNKI, PubMed, Web of Science, and PubChem) and the Traditional Chinese Medicine Systems Pharmacology Database and Analysis Platform (TCMSP, https://tcmsp.com/) database were obtained. The database included a network of chemicals, targets, and drug targets, as well as the pharmacokinetic properties of natural compounds with respect to oral bioavailability, drug similarity, intestinal epithelial permeability, the blood–brain barrier, and water solubility (He et al., 2022). Those meeting certain ADME properties and drug-likeness standards (OB ≥ 0.3, DL≥ 0.18) were chosen for further target prediction analysis (Bickerton et al., 2012). After filtering information, the component and target information of the four typical “hot” property herbs were collected including, FZ (21–28), GJ (5–48), RG (10–113), and WZY (30–454). All the chemical information was then used as a data source for target prediction. And then, the target of hypothyroidism was obtained from OMIM (https://www.omim.org/), Drug bank, and Gene Card (https://www.genecards.org/). Moreover, the intersection of the four typical “hot” property herb targets and hypothyroidism-associated targets were analyzed by a Venn Diagram (http://bioinformatics.psb.ugent.be/webtools/Venn/). And, the PPI network was performed on the STRING database (https://string-db.org/cgi/input.pl). The STRING database is used for searching known and predicted interactions between proteins. In addition to containing experimental data, it contains text mining results from PubMed abstracts, the integration of other database data, and it also uses bioinformatics methods to determine the predicted result. There are already many protein interaction databases, and STRING covers the most species among them. The specific settings in this system are as follows: select “*Homo sapiens*” in the organism column, select “evidence” in the meaning of network edges column, set the confidence to >0.9, remove the free protein, obtain the correlation data between the targets, and import the acquired data into Cytoscape 3.7.2 for the PPI network (He et al., 2022). The analyses of the GO functional annotation and KEGG signaling pathways were conducted by the Database for the Annotation, Visualization and Integrated Discovery (DAVID) v 6.8 (https://david.ncifcrf.gov/home.jsp) for Gene Ontology (GO) and KEGG pathway enrichment analyses, which were mainly involved in the molecular function (MF), biological process (BP), and cellular components (CC). The cut-off criterion of the DAVID analysis was *p* < 0.05, which was considered statistically significant (Li et al., 2021; Zhang et al., 2022).

### Statistical analysis

All the data were analyzed by using the SPSS 20.0 software (IBM; NY, United States). Statistical significance (*p* < 0.05) was assessed in comparison with the respective control for each experiment using one-way analysis of variance.

## Results

### Phenotypical analysis of the four typical “hot and cold” property herbs effect on hypothyroidism model

**FIGURE 1:**
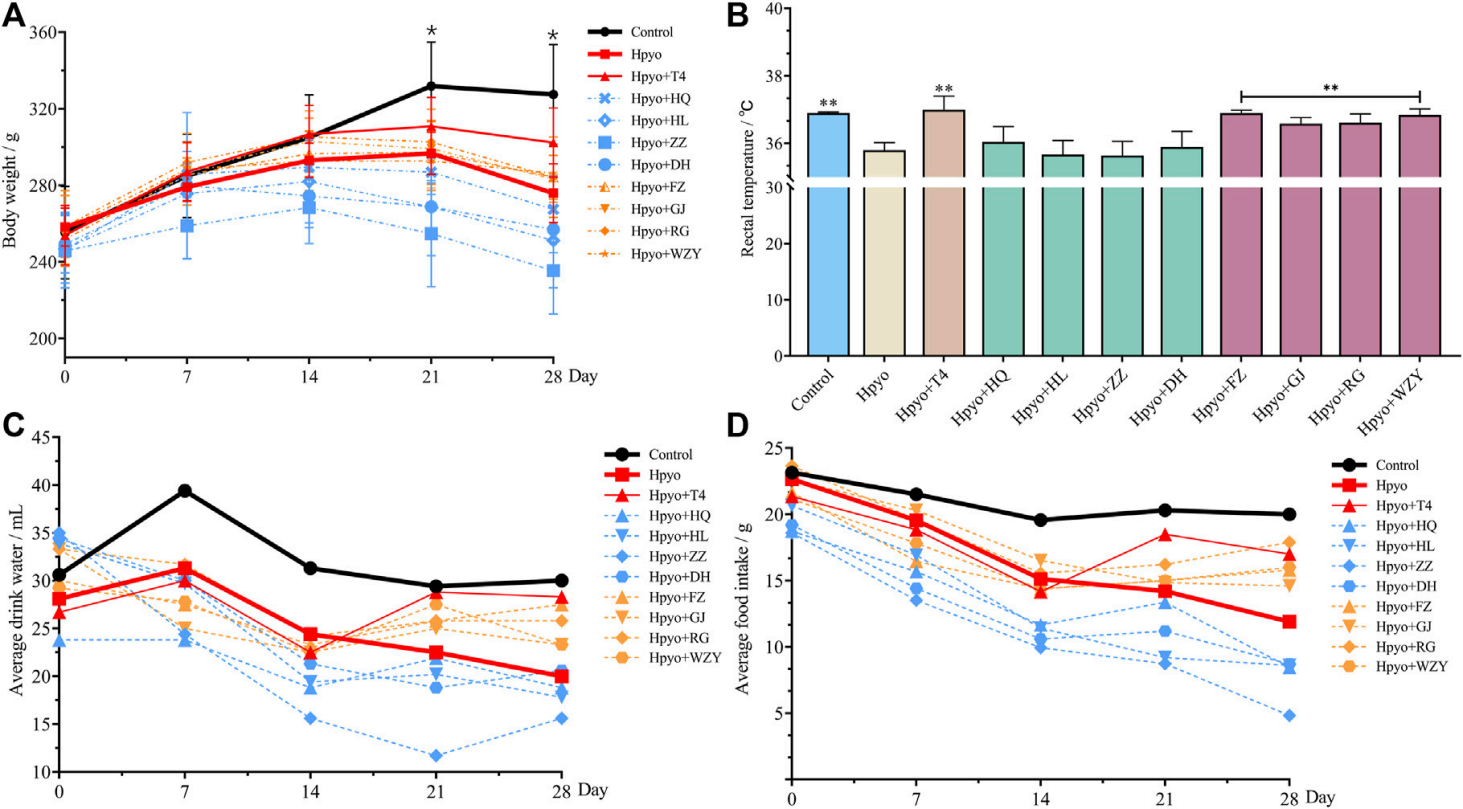
The altered trend of body weight **(A)**, body temperature **(B)** and average water **(C)**/food **(D)** intake in each group (n = 8). Note: Compared with the hypothyroidism model group, *p < 0.05, **p < 0.01.

Bodyweight, levels of food and water intake, and rectal temperature were important phenotypical indexes for hypothyroidism generally being evaluated. As shown in Figure 1, decreased phenotypical indexes were observed in hypothyroidism model rats, which suggested that the disordered metabolism occurred in the body of hypothyroidism rats. Compared with the hypo group, body weight, levels of food and water intake, and rectal temperature were ameliorated by the four typical “hot property” herbs on day 28 and show a similar effect. However, after administration of the four typical “cold” property herbs’ extraction for 4 weeks, the levels of food and water intake was less than the levels of the hypo group (Figures 1C,D), and the bodyweight loss and rectal temperature was not improving, compared with the hypo group (Figures 1A,B).

### Thyroid function assessment

**FIGURE 2:**
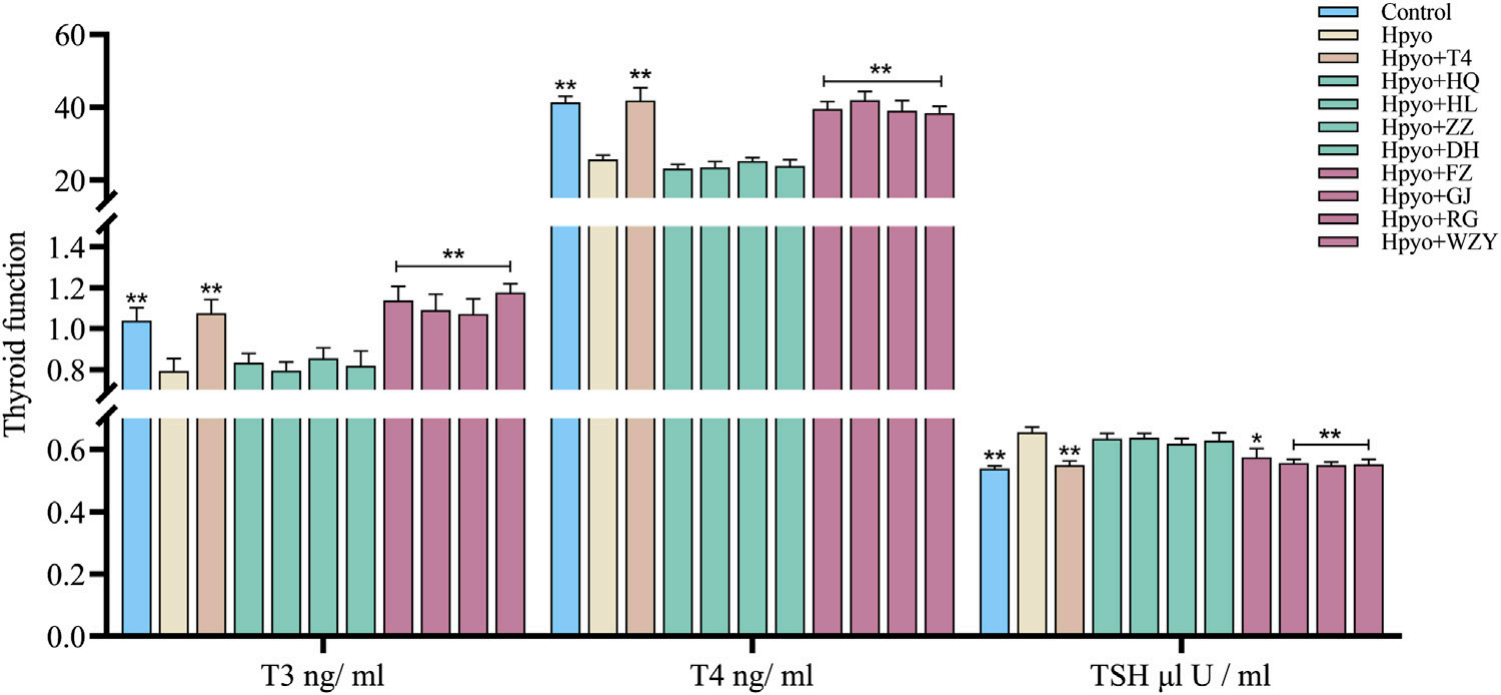
Effects of the four typical “hot” property herbs and the four typical “cold” property herbs on the thyroid function of the hypothyroidism model (*n* = 8). Note: Compared with the model group, *p < 0.05, **p < 0.01.

Firstly, the four typical “cold” property herbs and the four typical “hot” property herbs that affected the thyroid function of hypothyroidism rats were assessed by the levels of T3, T4, and TSH in the serum. As shown in Figure 2, after 4 weeks induced by the PTU, the levels of T3 and T4 were markedly decreased, and the concentration of TSH was obviously increased in the hypo group. However, the low levels of T3 and T4 and the elevated TSH concentration in the serum were significantly ameliorated by the four typical “hot” property herbs and showed similar intervention effects among them. This result suggested that the four typical “hot” property herbs possessed substantial treatment effects on the hypothyroidism rat model. While the four typical “cold” property herbs showed no improvement of the thyroid function in the hypothyroidism model.

### Metabolic profile analysis of the four “hot and cold” property herb effects on the hypothyroidism model

**FIGURE 3:**
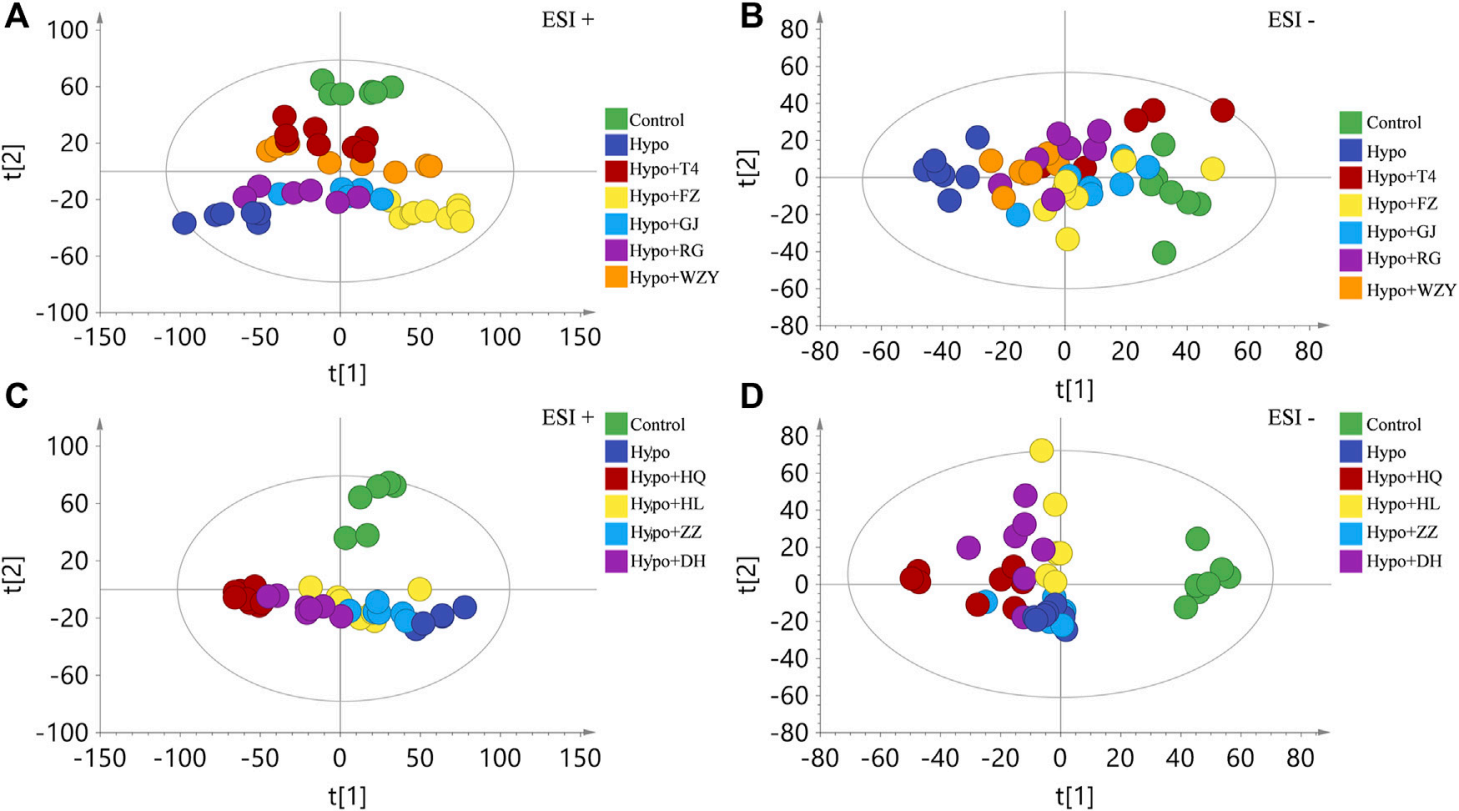
The principal component analysis (PCA) analysis of the four typical “hot” property herbs **(A,B)** represent in the positive and negative ion modes of the ESI, respectively) and the four typical “cold” property herbs **(C,D)** represent in the positive and negative ion modes of the ESI, respectively) effect on the hypothyroidism rats’ metabolic profile (*n* = 8).

To maximize collected the metabolism information and fingerprints about the hypothyroidism model, urine samples were acquired both in the positive and negative modes of the ESI (electron spray ionization). Firstly, an unsupervised principal component analysis (PCA) was carried out to explore the influences of the four typical “hot and cold” property herbs on the hypothyroidism model metabolic profile. As the result shows in Figure 3A, a clear difference was observed between the control group and the hypo group, suggesting that the metabolism state of the whole body was changed after 4 weeks induced by the PTU. After administering L-thyroxine and four typical “hot” property herbs, the metabolism state was away from the place of the hypothyroidism model state, and closer likely to the control group metabolism states in the positive ion mode. Meanwhile, the four typical “hot” property herbs also showed different extent improvement effects for hypothyroidism rats in the negative ion mode (Figure 3B). However, the results of the four typical “clod” property herbs affected the hypothyroidism model (as shown in Figures 3C, D), the four typical “cold” property herbs gathered together with the hypo group, or even further away from the control group than the hypothyroidism rats. In addition, the PCA score scatter plots between the control group and each “cold and hot” herb group is shown in the Supplementary Figures S1, S2A–J and all the grouping trends were clear. This result indicated that the “hot” herbs with the same property had similar effects, and herbs with opposite properties had different effects.

In the present study, combining the results of physical signs, thyroid function, and metabolic profile, we can conclude that the four typical “cold” property herbs showed no benefit in the hypothyroidism model. Therefore, the four typical “hot” property herbs that affected hypothyroidism were used for further mechanism analysis.

### Effects of the four typical “hot” property herbs on the histopathological changes in the thyroid tissue of hypothyroidism rats

**FIGURE 4:**
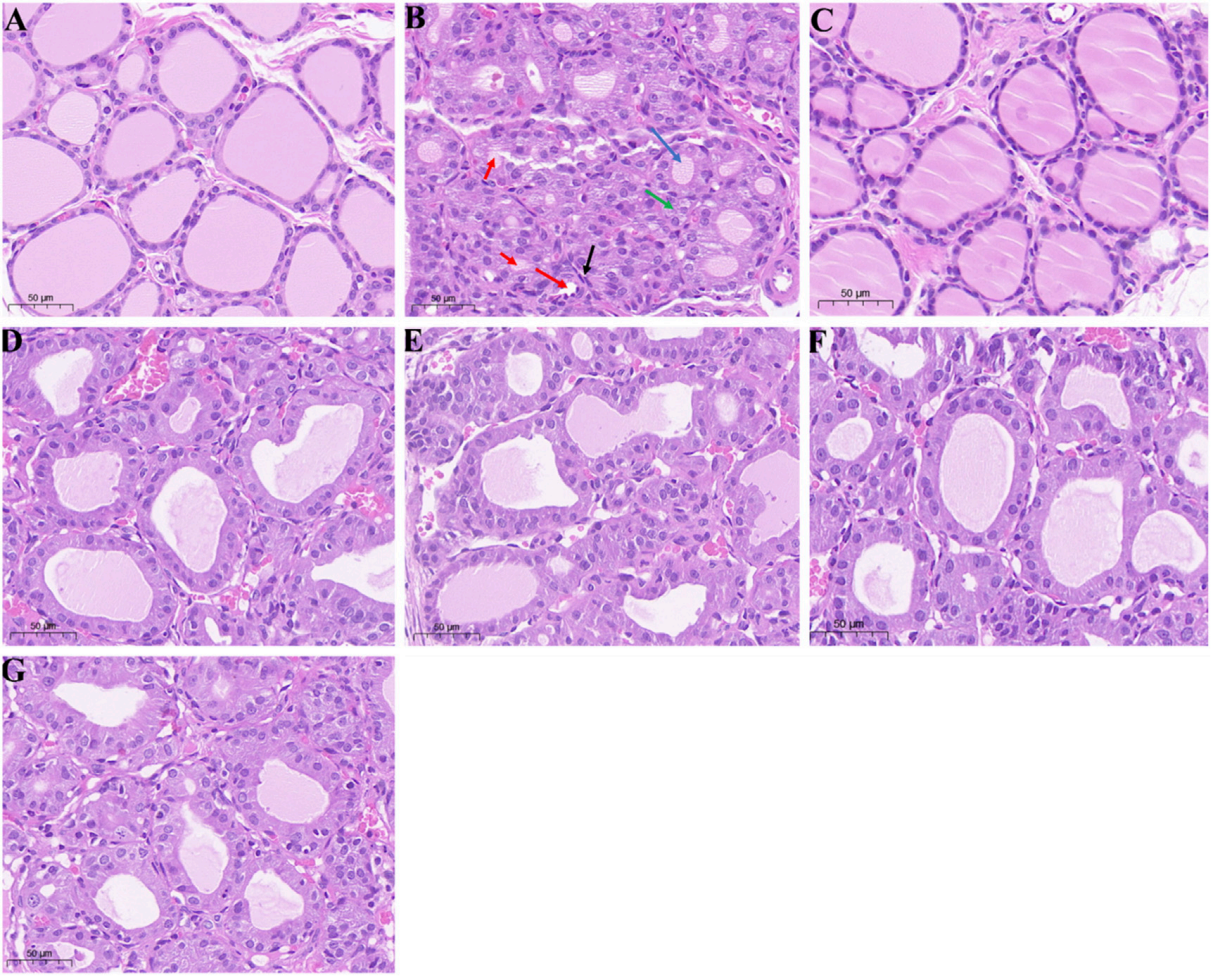
Histological examination (H and E) staining of thyroid glands; **(A–G)** represented the groups of control, hypo, T4, FZ, GJ, RG, and WZY, respectively. Magnification: ×20; Scale bar: 50 μm.

The effect of the four typical “hot property” herbs on the histopathological changes in the thyroid tissue of hypothyroidism rats is shown in Figure 4. In the control group, the thyroid gland performed a normal distribution, morphology, and architecture of follicles (Figure 4A). As shown in Figure 4B, clear thyroid gland pathological damage, including follicular luminal obliteration (as indicated by the red arrow), fibroblast proliferation (as indicated by the black arrow), hyperplasia (as indicated by the green arrow), and the follicles appearing with a smaller size (as indicated by the blue arrow), was observed in the hypo group. However, these PTU-induced morphological changes were attenuated by the administration of the extraction of the four typical “hot” property herbs (as shown in Figures 4D–G).

### The potential biomarker identification and cluster analysis

**FIGURE 5:**
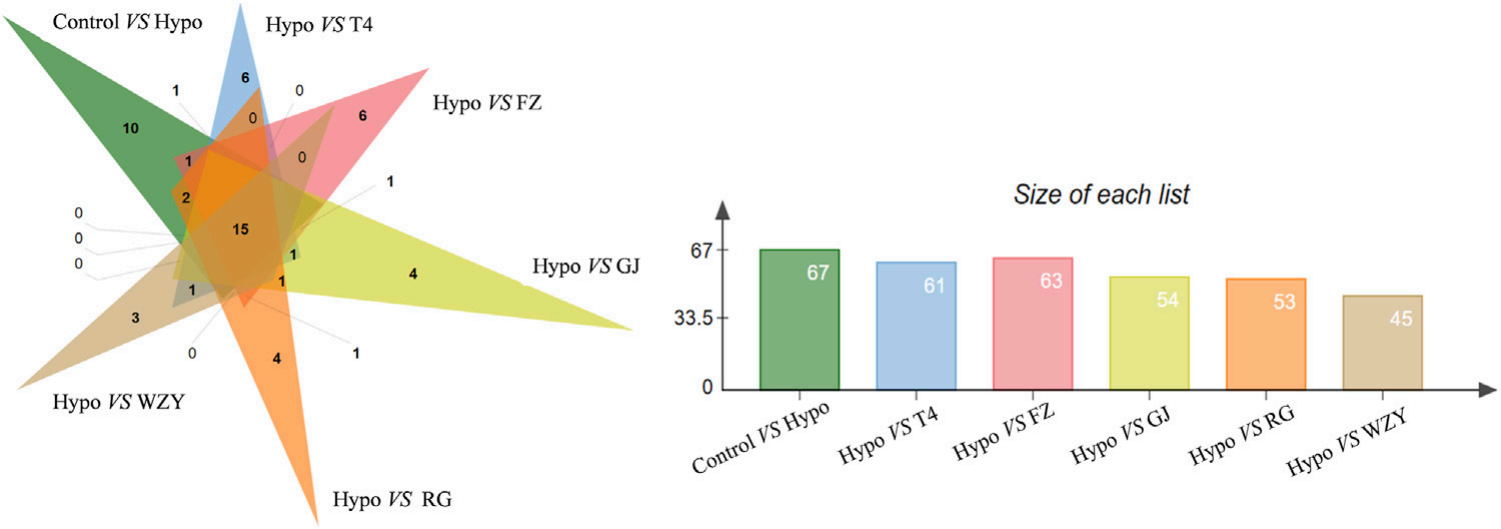
The Veen analysis of potential biomarkers in the four typical “hot” property herb groups.

As a supervised learning approach, OPLS_DA could filter out non-essential variables and remarkably improve the accuracy of classification (Zuo et al., 2018). As the results showed, the OPLS_DA score plots between the control group and each hot herb group were significantly separate from the hypothyroidism model (Supplementary Figures S3, S4). Meanwhile, the potential biomarkers were obtained from the OPLS-DA analysis with the predetermined rules (VIP>1; *p* <0.05). It was suggested that the metabolism state or metabolites have altered. Combined with the results of PCA, those changed metabolites could be the key potential biomarker of working mechanisms. In this study, a total of common 15 specific metabolic biomarkers (Figure 5), related both to hypothyroidism models and the four typical “hot” property herbs, were identified by the QI software (as shown in Table 1).

**TABLE 1:** The biomarkers are affected by the four typical “hot” property herbs.

Metabolite	KEGG ID	Adducts	Formula	m/z	RT (min)	a	b	b	b	b	b
						Hypo	Hypo + T4	Hypo + FZ	Hypo + GJ	Hypo + RG	Hypo + WZY
Indole acetaldehyde	C00637	M + H	C10H9NO	160.0762	10.02	↑	↓	↓	↓	↓	↓
5-Thymidylic acid	C00364	M + H	C10H15N2O8P	323.0631	11.49	↑	↓	↓	↓	↓	↓
L-Phosphoarginine	C05945	M + Na	C6H15N4O5P	277.0668	13.07	↑	↓	↓	↓	↓	↓
Xanthurenic acid	C02470	M + H-H2O	C10H7NO4	188.0349	14.39	↑	↓	↓	↓	↓	↓
Indole pyruvate	C00331	M + H-H2O, M + H	C11H9NO3	204.0660	14.39	↑	↓	↓	↓	↓	↓
Nicotinamide ribotide	C00455	M + Na	C11H16N2O8P+	358.0552	6.92	↑	↓	↓	↓	↓	↓
Betaine	C00719	M + Na	C5H11NO2	140.0687	9.16	↑	↓	↓	↓	↓	↓
Diadenosine tetraphosphate	C01260	M-H2O-H, M-	C20H28N10O19P4	817.0266	19.59	↓	↑	↑	↑	↑	↑
2-Oxoarginine	C03771	2M-H	C6H11N3O3	345.1543	3.42	↑	↓	↓	↓	↓	↓
Deoxyuridine	C00526	M + HCOO-	C9H12N2O5	273.0741	6.26	↑	↓	↓	↓	↓	↓
L-Histidinol	C00860	2M-H	C6H11N3O	281.1733	8.88	↑	↓	↓	↓	↓	↓
Uridine	C00299	M-H, 2M-H	C9H12N2O6	243.0618	0.92	↓	↑	↑	↑	↑	↑
2-Phenylethanol	C05853	M + H-H2O	C8H10O	105.0704	11.11	↑	↓	↓	↓	↑	↑
Prostaglandin H2	C00427	M-H	C20H30O5	349.2021	9.25	↓	↑	↓	↓	↑	↓
Nicotinate D-ribonucleoside	C05841	M-	C11H14NO6+	257.0817	10.41	↑	↑	↑	↑	↓	↑

a Represent compared with the control group.

b Represent compared with the model group.

**FIGURE 6:**
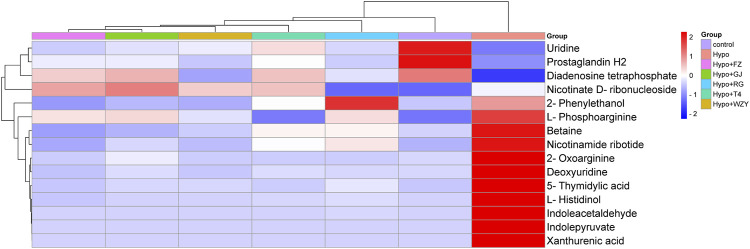
The heat map and cluster analysis for the potential biomarker.

The hot map and cluster analysis results for those metabolites in the control, hypothyroidism model, L-thyroxine (T4), and the four typical “hot” property herb groups not only responded to the importance of metabolites but also validated the previous analysis results. As shown in Figure 6, the result of a cluster analysis shows that the control group is grouped with the L-thyroxine, RG, WZY, GJ, and FZ groups in turn, and is ultimately related to the Hypo group. This result further revealed the improvement effects of the four typical “hot” property herbs on hypothyroidism rats.

### The pathway enrichment analysis and metabolic network

**FIGURE 7:**
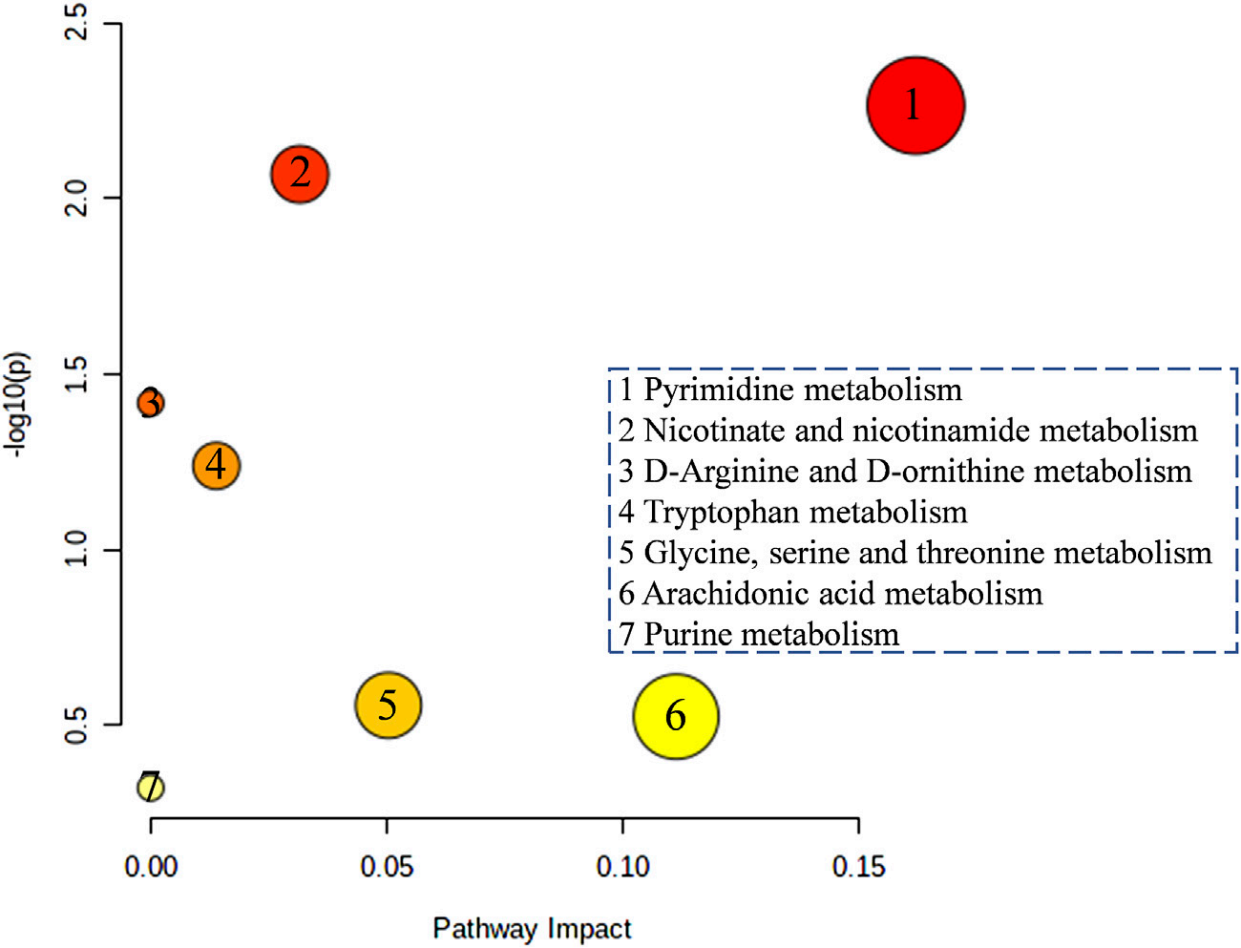
The function enrichment analysis of the four typical “hot” property herbs affected by the hypothyroidism model.

In order to further analyze how the four typical “hot” property herbs work on the hypothyroidism model by those different potential biomarkers, the altered metabolites were annotated by the KEGG database, and the pathway was analyzed by the Metabo-Analyst. It was found that 7 perturbed metabolism pathways were involved (Figure 7), mainly including pyrimidine metabolism, nicotinate and nicotinamide metabolisms, D-arginine and D-ornithine metabolisms, tryptophan metabolism, Glycine, serine and threonine metabolisms, arachidonic acid metabolism, and purine metabolism.

### Network analysis of the four “hot” property herbs and hypothyroidism model

**FIGURE 8:**
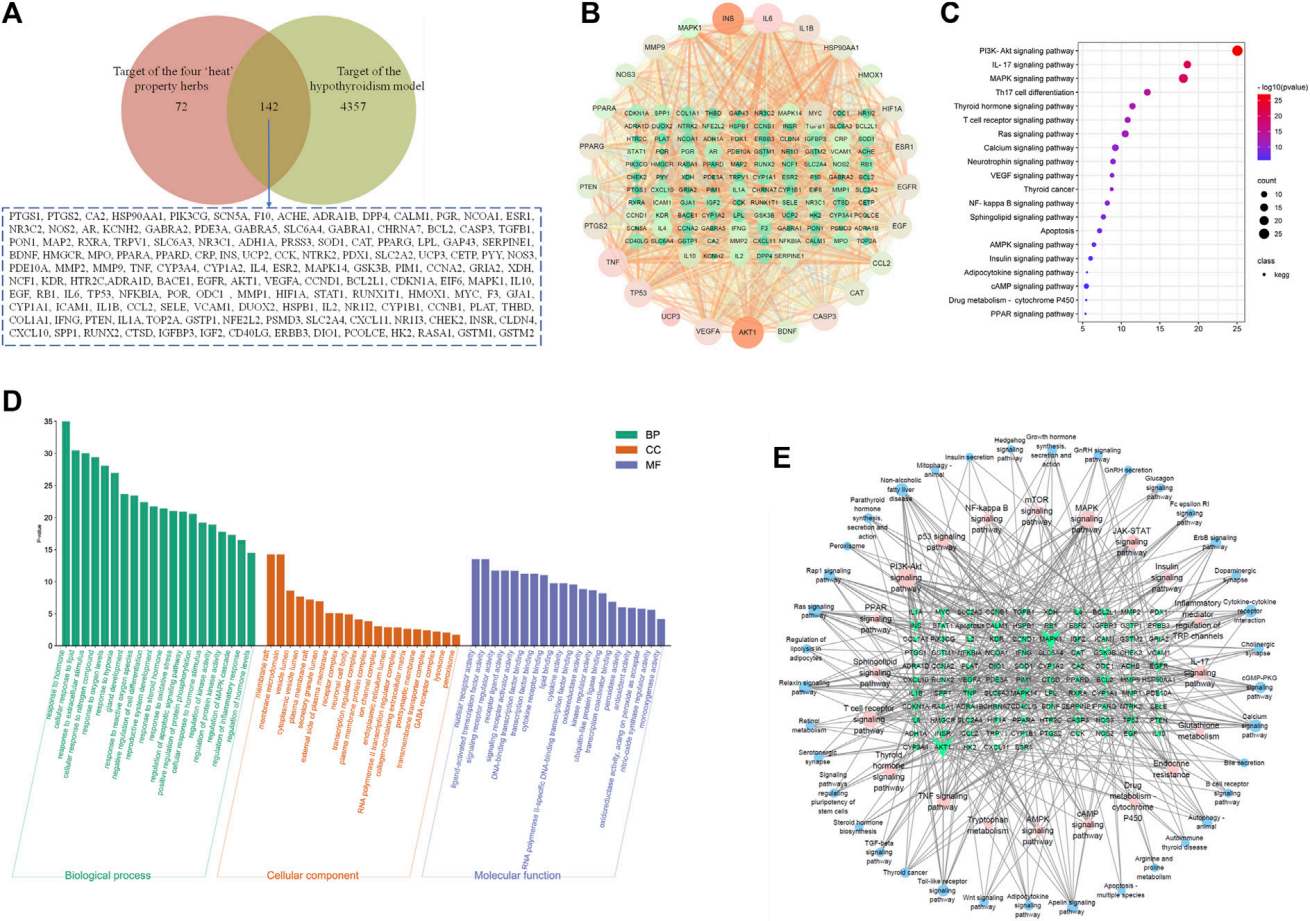
The target and network analysis of the four typical “hot” property herbs and the hypothyroidism model. **(A)**: The Venn diagram of the common targets obtained from hypothyroidism and components of the four typical “hot” property herbs; **(B)**: The PPI network with high-frequency node analysis by 142 common targets; **(C)**: The common target pathway enrichment analysis; **(D)**: the GO analysis of the biological processes, cellular components, and molecular functions with common targets; **(E)**: The key targets and signaling pathway network relation-based GO and KEGG analyses.

Then, the potential ingredients and molecular targets of the four typical “hot” property herbs were performed, based on a network pharmacological approach. We identified 214 potential targets associated with the four typical “hot” property herbs and 4,499 targets associated with hypothyroidism, and the common targets of 142 were selected as the key targets (Figure 8A). In combination with high-frequency node analysis in the PPI network (Figure 8B), we found that 25 targets with a circular layout and dark color, including INS (insulin), IL6 (interleukin 6), MAPK (mitogen-activated protein kinase), VEGFA (vascular endothelial growth factor A), TNF (tumor necrosis factor), AKT1 (protein kinase B1), and PPARs (peroxisome proliferator-activated receptors) could the key target proteins. In addition, the GO and KEGG pathway analyses were conducted by the STRING database for the pathway enrichment and function analysis based on the potential targets. The molecular function analysis revealed that these putative targets were mainly involved in the response to the hormones, lipids, and reactive oxygen species, and adjust to the MAPK cascade, inflammatory response, and oxidative stress biological process (as shown in Figure 8D). Moreover, the molecular function analysis revealed that those targets not only modulated the nuclear receptor activity, signaling receptor regulator activity, kinase regulator activity, oxidoreductase activity, peroxidase activity, and monooxygenase activity, but also tuned DNA-binding transcription factor binding, lipid binding, and cytokine receptor binding. Those biological processes and molecular functions related to the pathway mainly included: the PI3K/Akt signaling pathway, IL-17 signaling pathway, MAPK signaling pathway, thyroid hormone signaling pathway, Ca^2+^ signaling pathway, VEGF signaling pathway, NF-κB signaling pathway, sphingolipid signaling pathway, AMPK signaling pathway, insulin signaling pathway, cAMP signaling pathway, cytochrome P450, and PPAR signaling pathway (as shown in Figure 8C). The network among the key targets and signaling pathways is shown in Figure 8E.

### The metabolomic and integrated analysis and verification

**FIGURE 9:**
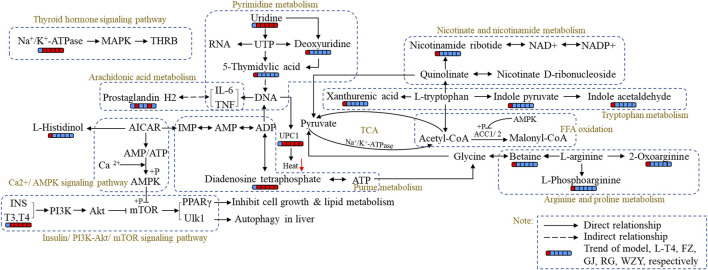
The metabolism network of the four typical “hot” property herbs influences the hypothyroidism model based on the hit target of the network analysis and the altered biomarker. Red represents the increased trend; blue represents the decreased trend.

**FIGURE 10:**
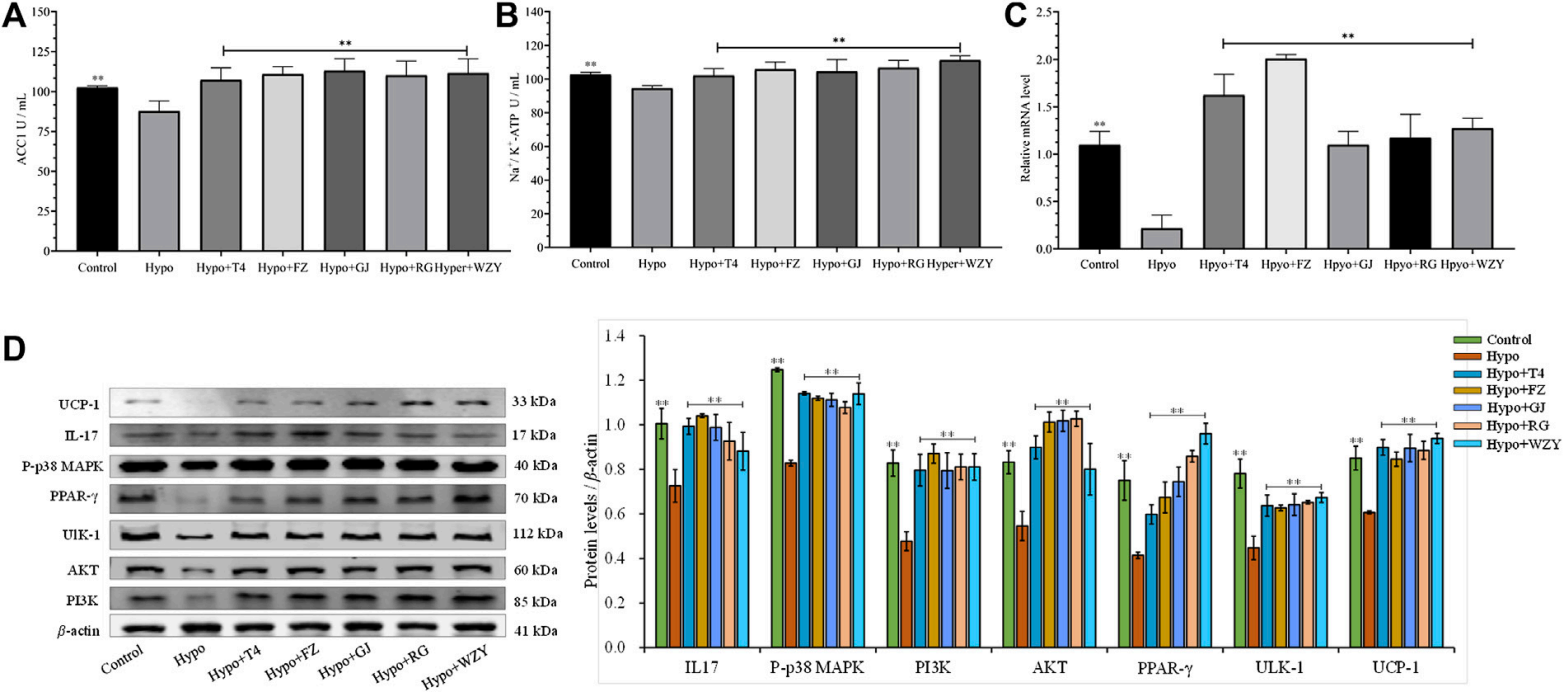
The levels of the ACC1 enzyme **(A)**, Na^+^/K^+^-ATP enzyme **(B)** (*n* = 8) in the liver and the real-time PCR analysis of mRNA levels of UCP-1 in BATs from the hypothyroid model rat using NADPH as the internal control **(C)**; the protein expression levels of IL17, MAPK, PI3K, Akt, and ULK-1 in the liver, and UCP-1 in BATs **(D)**.

In this study, the perturbed metabolism network, based on the found metabolism pathway by the Metabo-Analyst and the network analysis, was constructed (Figure 9). This clearly illustrated the relationship among disturbed metabolic pathways, recalled metabolites, and key target proteins. As Figure 9 shows, the PI3K- Akt signaling pathway, Ca^2+^- AMPK signaling pathways are associated with lipid metabolism and energy metabolism; tryptophan metabolism, nicotinate, and nicotinamide metabolism, and arginine and proline metabolisms were associated with energy metabolism and thyroid hormone levels. Moreover, the mRNA level of UCP-1 in BAT, the levels of the Na^+^/K^+^-ATP enzyme, and ACC1 in the liver were detected to verify the network analysis and metabolomics results. As shown in Figure 10, decreased levels of UCP-1, the Na^+^/K^+^-ATP enzyme, and ACC 1 were observed, which suggested the presence of an energy metabolism imbalance and a disordered lipid metabolism in hypothyroidism rats. And, the four typical “hot” property herbs could ameliorate this disordered energy metabolism and lipid metabolism. Moreover, key proteins (as shown in Figure 10D) in the PI3K/Akt signaling pathway and thyroid hormone signaling pathway were Western blot analyzed and validated, which were restored by the four typical “hot” property herbs included IL17, MAPK, PI3K, Akt, and ULK-1.

## Discussion

### The curative effect of the four typical “hot” property herbs on the hypothyroidism model

Hypothyroidism, an endocrine disorder disease, is known as the classic cold syndrome category in Chinese medicine (Chen et al., 2021). The medication principle of “treating cold/heat syndrome with hot/cold herbs” is widely used and affirmed in the clinical practice of Chinese medicine. And previous experiments in our laboratory have reported that a typical “hot” herb (*Aconiti lateralis radix praeparaia*) could improve hypothyroidism in rats with cold syndrome by adjusting the substances and energy metabolism (Xiao et al., 2017). In this study, a series of typical clinical features of hypothyroidism was observed in the hypo group, including huddling up together inactively, struggling weakly during catching, less water and food intake, and a rectal temperature decrement (Zhou et al., 2019). In addition, the decreased levels of T3 and T4, increased TSH concentration, as well as a damaged thyroid tissue also confirmed the success of model building (Ahmed et al., 2010). And the slight weight loss was explained by the lower growth-stimulating effect of thyroid hormones or the loss of appetite due to PTU (Müller and Seitz, 1981). Brown adipose tissue (BAT), as a major thermogenic tissue, is dominated by the thyroid hormones and the nervous system (Obregon, 2014). The uncoupling protein-1 (UCP-1), expressed in the BAT mitochondria, can release energy in the form of heat, rather than storage in the ATP. In this study, a decreased gene expression of UCP1, rectal temperature, and the Na^+^/K^+^-ATP enzyme were also observed in the hypothyroidism model (Figure 10). So, the energy metabolism and thermogenics were inhibited in hypothyroidism rats, and this declined the metabolism status that could be improved by the four typical “hot” property herbs.

As shown in the present studies, the results of physical signs, thyroid function, the histological evaluation of thyroid glands, and the metabolism profile analysis clarified that the four typical “hot” property herbs had therapeutic effects on the hypothyroidism model (a cold syndrome). But the four typical “cold” herbs were shown to have no benefit, which was consistent with the TCM theory. In the metabolism profile analysis, the QC sample was closely gathered together in the PCA score scatter plots both in the positive and negative modes, which suggested the great stability of the instrument and the acquiring methods (Supplementary Figures S1A, S2A). Meanwhile, the four typical “hot” property herbs were closely gathered together with the control group, and far away from the hypo group in the PCA score scatter plots (Figures 3A,B) and heat maps (Figure 6) which suggests that the “hot” herbs with the same properties had similar effects and herbs with opposite properties had different effects. In addition, the different curative among the four typical “hot” property herbs mainly contributed to the contents of the metabolites. The metabolism network (Figure 9) and network analysis (Figure 8) further elucidated the inner mechanism.

### Influence of the four typical “hot” property herbs on lipid metabolism

Evidence has reported the direct effects of thyroid hormones on hepatic lipid metabolism (Sinha et al., 2018) and the high cardiovascular disease risk in hypothyroidism (Ermantas et al., 2010), which were largely attributed to disordered lipid metabolism. As we all know, the process of converting glucose to fatty acids termed, “*de novo* lipogenesis,” is induced by the thyroid hormone, and then produces fatty acids, triacylglycerol, and VLDL (Liang et al., 2011). ACC1 is a key enzyme in lipid synthesis, regulated by thyroid hormones (Lu et al., 2011). Studies have reported in hypothyroidism suggesting that lipid synthesis was reduced in the liver, due to the decreased activity of hepatic lipase, which can be recovered with thyroid hormone replacement therapy (Brenta et al., 2016). And a high level of T3 has been used for the treatment of hypercholesterolemia and obesity (Krotkiewski, 2002). In this study, a lower enzymatic activity of ACC1 and a decreased level of acetyl-CoA were observed in hypothyroidism livers (Figure 9), suggesting that the lipid metabolism and energy metabolism were disrupted in hypothyroidism, which is consistent with the previous study. Moreover, the ACC1 and acetyl-CoA were significantly upregulated by the four typical “hot” property herbs. These results suggest that the four typical “hot” property herbs could promote lipid metabolism, which might be one of the mechanisms of “hot” property herbs to improve hypothyroidism.

Current research shows that the mode of action of thyroid hormones mainly include nuclear transcriptional and non-genomic mechanisms (Cao et al., 2005), such as (ⅰ) the transcriptional regulation of target genes via THRs binding to TREs and the recruitment of co-activators to increase RNA polymerase binding to the basal transcriptional protein complex (Sinha et al., 2018). (ⅱ) Thyroid hormones could regulate hepatic lipid metabolism via activating the PI3K - Akt (RACα serine/threonine-protein kinase) signaling pathway, the PKA (cAMP-protein kinase A) and the Ca^2+^- AMPK signal pathways (Yamauchi et al., 2008), which is a non-genomic regulatory mechanism. In metabolomic and network enrichment analyses, the PPI network and metabolism network analyses (as shown in Figures 8, 9), the PI3K - Akt signaling pathway, and the Ca^2+^- AMPK signal pathways were closely associated with purine metabolism, pyrimidine metabolism, histamine metabolism, energy metabolism, and lipid metabolism. And there is evidence that purine metabolism is closely related to hypothyroidism (Wu et al., 2013). In addition, a decreased level of diadenosine tetraphosphate in purine metabolism and increased TSH concentration were detected in hypothyroidism rats, which suggests that diadenosine tetraphosphate is involved in the synthesis of TSH. This result was supported by previous studies (Friedrich et al., 2017). So, the four typical “hot” property herbs may ameliorate the hypothyroidism status by the PI3K- Akt signaling pathway, Ca ^2+^- AMPK signaling pathways for promoting lipid metabolism, energy metabolism through, as well promoting TSH synthesis, purine metabolism.

### Influence of the four typical “hot” property herbs on tryptophan metabolism and energy metabolism

Tryptophan is an essential amino acid, involved in a variety of metabolisms, such as energy metabolism (Zhou et al., 2019) and thyroid hormone metabolism (Pereira et al., 2017). It is reported that a dietary tryptophan deficiency can alter the thyroid hormone levels (Vaccari et al., 1990) and lightened depression or anorexia and a poor appetite can be caused by low thyroid hormone levels (Pereira et al., 2017). Moreover, approximately 95% of tryptophan is metabolized via the kynurenine pathway and produced into xanthurenic acid, indole acetaldehyde, and indole pyruvate, which are crucial cofactors in glycolysis and mitochondrial respiration (Zhou et al., 2019), and are involved in the TCA cycle in the form of acetyl-CoA (as shown in Figure 9). So, tryptophan metabolism is closely associated with glycolysis, mitochondrial respiration, and thyroid hormone metabolism. As shown in Figure 9, it was observed in our study that xanthurenic acid, indole acetaldehyde, and indole pyruvate, the downstream metabolic products of tryptophan, clearly increased in the hypo group. The aberrantly increased catabolism of tryptophan may worsen the lack of tryptophan *in vivo*, and this change was consistent with those previously reported (Wu et al., 2013), and then result in thyroid dysfunction (Kulikov et al., 1999). Meanwhile, the decrease in the acetyl-CoA level detected by the ELISA kit has corroborated this result. However, after the four typical “hot” property herbs were administered to hypothyroidism rats, the levels of xanthurenic acid, indole acetaldehyde, and indole pyruvate returned to normal (Figure 9). Correspondingly, the symptoms of rectal temperature, water and food intake, and the mental state were healed as well. Therefore, the four typical “hot” property herbs could ameliorate the disordered tryptophan metabolism in hypothyroidism rats, thus promoting the lower energy metabolism and thyroid hormone levels.

In summary, the four typical “hot” property herbs improve hypothyroidism in rats possibly through the PI3K-Akt signaling pathway, Ca ^2+^- AMPK signaling pathways to promote lipid metabolism, energy metabolism, and tryptophan metabolism to improve the declined energy metabolism and thyroid hormone levels.

## Conclusion

In this study, we firstly reported that all of the four typical “hot” property herbs could ameliorate the hypothyroidism model to different degrees, whereas no significant amelioration effect was noted in the four typical “cold” property herbs. Moreover, a metabolomic strategy combined with network analysis was successfully performed and well interpreted the mechanism of the four typical “hot” property herbs on hypothyroidism based on the theory of “cold and hot” properties of the TCM. Specifically, the four typical “hot” property herbs might influence hypothyroidism by (ⅰ) acting on the PI3K-Akt signaling pathway, Ca ^2+^- AMPK signaling pathways to promote lipid metabolism and energy metabolism; (ⅱ) promoting thyroid hormone synthesis through purine metabolism; (ⅲ) acting on the tryptophan metabolism to improve the declined energy metabolism and thyroid hormone levels. These common potential biomarkers might reflect the characteristics of the body after the administration of the four typical “hot” property herbs, in other words, these biomarkers represent the “hot” property of Chinese medicines.

## Data Availability

The original contributions presented in the study are included in the article/Supplementary Material; further inquiries can be directed to the corresponding authors.
