# Supplementary material for: Application of metabolomics and network analysis to reveal the ameliorating effect of four typical “hot” property herbs on hypothyroidism rats
Source: Front Pharmacol. 2022 Aug 25;13:955905. doi: 10.3389/fphar.2022.955905 (PMC9452843; doi:10.3389/fphar.2022.955905)
Supplement: Supplementary file 1 [file DataSheet1.docx]

Figure S 1 The PCA score scatter plots between the control group (1), Hypo group (12) and each individual cold and hot herbs groups (number in the picture:T4(13), HQ (14), HL(15), ZZ(16), DH(17); FZ(18), GJ(19), RG(20), WZY(21)) in positive ion source mode. (A: control *vs.* Hypo *vs.* QC; B: control *vs.* Hypo *vs.* Hypo +T4; C: control *vs.* Hypo *vs.* Hypo + FZ; D: control *vs.* Hypo *vs.* Hypo + GJ; E: control *vs.* Hypo *vs.* Hypo + RG; F: control *vs.* Hypo *vs.* Hypo + WZY; G: control *vs.* Hypo *vs.* Hypo + HQ; H: control *vs.* Hypo *vs.* Hypo + HL; I: control *vs.* Hypo *vs.* Hypo +ZZ; J: control *vs.* Hypo *vs.* Hypo + DH.)

Figure S 2 The PCA score scatter plots between the control group (1), Hypo group (12) and each individual cold and hot herbs groups (number in the picture:T4(13), HQ (14), HL(15), ZZ(16), DH(17); FZ(18), GJ(19), RG(20), WZY(21)) in negative ion source mode. (A: control *vs.* Hypo *vs.* QC; B: control *vs.* Hypo *vs.* Hypo +T4; C: control *vs.* Hypo *vs.* Hypo + FZ; D: control *vs.* Hypo *vs.* Hypo + GJ; E: control *vs.* Hypo *vs.* Hypo + RG; F: control *vs.* Hypo *vs.* Hypo + WZY; G: control *vs.* Hypo *vs.* Hypo + HQ; H: control *vs.* Hypo *vs.* Hypo + HL; I: control *vs.* Hypo *vs.* Hypo +ZZ; J: control *vs.* Hypo *vs.* Hypo + DH.)

Figure S 3. The OPLS_ DA analysis, S-plot score, and Coefficients vs. VIP of compared between the control group *vs*. the Hypo group (A), the Hypo group *vs*. the Hypo+T4 group (B), the Hypo group *vs*. the Hypo+FZ group (C), the Hypo group *vs*. the Hypo+GJ group (D), the Hypo group *vs*. the Hypo+RG group (E), the Hypo group *vs*. the Hypo+WZY group (F), in the positive ion mode.

Figure S 4. The OPLS_ DA analysis, S-plot score, and Coefficients vs. VIP of compared between the control group *vs*. the Hypo group (A), the Hypo group *vs*. the Hypo+T4 group (B), the Hypo group *vs*. the Hypo+FZ group (C), the Hypo group *vs*. the Hypo+GJ group (D), the Hypo group *vs*. the Hypo+RG group (E), the Hypo group *vs*. the Hypo+WZY group (F), in the negative ion mode.
